# Bidirectional Scaling of Astrocytic Metabotropic Glutamate Receptor Signaling following Long-Term Changes in Neuronal Firing Rates

**DOI:** 10.1371/journal.pone.0049637

**Published:** 2012-11-15

**Authors:** Alison X. Xie, Min-Yu Sun, Thomas Murphy, Kelli Lauderdale, Elizabeth Tiglao, Todd A. Fiacco

**Affiliations:** 1 Graduate Program in Neuroscience, University of California Riverside, Riverside, California, United States of America; 2 Graduate Program in Cellular, Molecular, and Developmental Biology, University of California Riverside, Riverside, California, United States of America; 3 Undergraduate Neuroscience Major, University of California Riverside, Riverside, California, United States of America; 4 Department of Cell Biology and Neuroscience and Center for Glial-Neuronal Interactions, University of California Riverside, Riverside, California, United States of America; Baylor College of Medicine, United States of America

## Abstract

Very little is known about the ability of astrocytic receptors to exhibit plasticity as a result of changes in neuronal activity. Here we provide evidence for bidirectional scaling of astrocytic group I metabotropic glutamate receptor signaling in acute mouse hippocampal slices following long-term changes in neuronal firing rates. Plasticity of astrocytic mGluRs was measured by recording spontaneous and evoked Ca^2+^ elevations in both astrocytic somata and processes. An exogenous astrocytic Gq G protein-coupled receptor was resistant to scaling, suggesting that the alterations in astrocyte Ca^2+^ signaling result from changes in activity of the surface mGluRs rather than a change in intracellular G protein signaling molecules. These findings suggest that astrocytes actively detect shifts in neuronal firing rates and adjust their receptor signaling accordingly. This type of long-term plasticity in astrocytes resembles neuronal homeostatic plasticity and might be important to ensure an optimal or expected level of input from neurons.

## Introduction

A number of studies *in situ* and *in vivo* has established strong evidence for neuron-to-astrocyte signaling in the brain [1]–[6]. While a primary target of synaptically-released neurotransmitter is ionotropic receptors in the postsynaptic membrane to produce shifts in neuronal membrane potential, neurotransmitter can spill out of the synapse to stimulate Gq G protein-coupled (metabotropic) receptors (Gq GPCRs) on perisynaptic astrocyte processes [1]–[6]. Gq GPCRs are coupled to a canonical signaling cascade that includes release of Ca^2+^ from IP_3_ receptor-sensitive internal stores. Therefore, recording astrocyte Ca^2+^ activity provides a very useful tool to measure the timing of astrocytic Gq GPCR activation.

Previous studies *in vitro* suggest that Ca^2+^ activity can also be used to estimate changes in Gq GPCR expression levels. A number of specific changes in spontaneous and evoked Ca^2+^ elevations occur following manipulation of Gq GPCR expression levels in cultured cells. These include an increase in the percentage of cells in the population exhibiting spontaneous Ca^2+^ elevations [7]–[9], a higher response probability to agonist stimulation [10], [11], and a decrease in Ca^2+^ response latency to agonist [10]–[12]. More recently, experiments in astrocytes *in situ* studied the relationship between agonist concentration and astrocytic Ca^2+^ response characteristics. With increasing agonist concentration, astrocytic Gq GPCR Ca^2+^ responses change from single-peak, to multi-peak, to long-lasting plateau patterns [7], [13]–[15]. An exciting possibility is that astrocytic Gq GPCRs exhibit plasticity in response to changes in neuronal activity *in situ*. Here, we set out to determine if long-term changes in CA3 neuron firing rates lead to long-term plasticity of spontaneous and evoked astrocytic Gq GPCR Ca^2+^ elevations in CA1 stratum radiatum (s.r.) astrocytes in acute hippocampal slices.

Based on a comprehensive analysis of evoked and spontaneous astrocyte Ca^2+^ elevations in the astrocyte soma and fine processes, here we provide evidence indicating bidirectional, rapid scaling of group I metabotropic glutamate receptor (mGluR) Ca^2+^ signaling in s.r. astrocytes of the hippocampus *in situ* following long-term increase or decrease in neuronal firing rates. Calcium responses evoked by stimulating a foreign astrocytic Gq GPCR (the MrgA1R) were unaltered, suggesting that changes in Ca^2+^ activity are due to changes in surface mGluR activity rather than components of the intracellular Gq GPCR signaling pathway. Taken together, our findings suggest that astrocytes scale their mGluR signaling activity bi-directionally following long-term changes in neuronal action potentials.

## Materials and Methods

### Ethics Statement

Experiments in this study were specifically approved by the Institutional Animal Care and Use Committee guidelines of the University of California, Riverside, to ensure minimal animal suffering.

**Figure 1:**
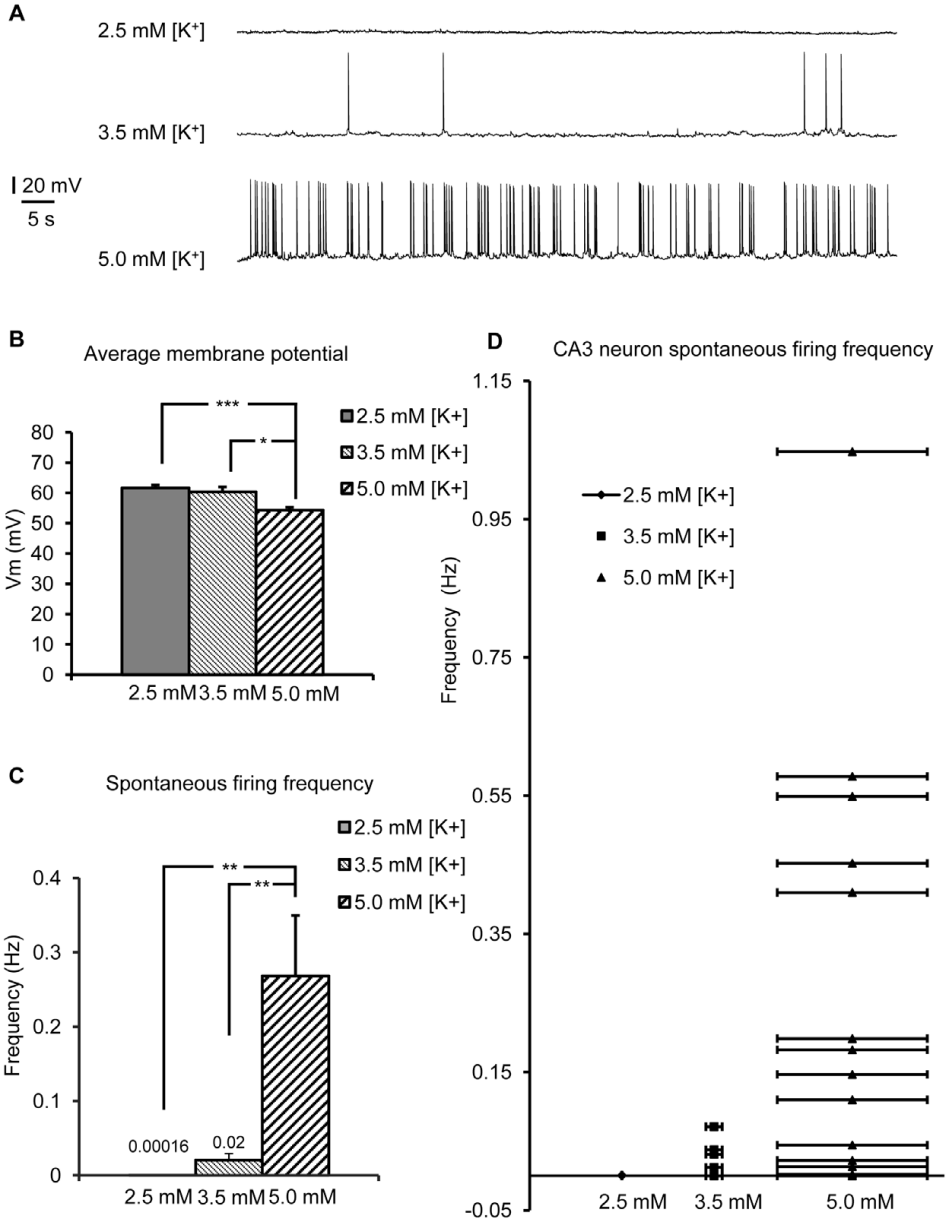
Firing rates of CA3 neurons in hippocampal slices as a function of [K^+^]_o_. (A) Whole-cell current clamp recording of CA3 neuron membrane potential indicated that CA3 neurons rarely fired spontaneous action potentials in 2.5 mM [K^+^]_o_ ACSF (n = 12), while in 3.5 mM [K^+^]_o_ ACSF, CA3 neurons fired occasional spontaneous APs (n = 8). CA3 neurons were significantly depolarized in 5 mM [K^+^]_o_ ACSF and regularly fired APs (n = 15). (B) There was a significant difference in membrane potential between CA3 neurons incubated in 2.5 mM [K^+^]_o_ ACSF *vs*. those incubated in 3.5 mM and 5.0 mM [K^+^]_o_ ACSF. Neurons became more depolarized with increasing [K^+^]_o_. (C) CA3 neurons incubated in 3.5 mM and 5.0 mM [K^+^]_o_ ACSF had more spontaneous action potentials than those incubated in 2.5 mM [K^+^]_o_ ACSF. (D) The distribution of frequency of spontaneous action potentials from individual CA3 neurons tested. In general, CA3 neurons incubated in 3.5 mM and 5.0 mM [K^+^]_o_ ACSF had more frequent spontaneous APs compared to those incubated in 2.5 mM [K^+^]_o_ ACSF.

### Preparation of Acute Hippocampal Slices

Acute slices were chosen for this study because the majority of the neuronal network and neuron-to-astrocyte communication remains intact in acute slices. It has been estimated that more than 40% of the synapses are still associated with astrocyte processes after recovery from dissection [16]. Acute mouse hippocampal slices were prepared as described previously (Fiacco et al. 2007). Twelve to eighteen-day-old C57BL/6J mice (Jackson Laboratory, Bar Harbor, ME) were anaesthetized using isoflurane and decapitated. Parasagittal hippocampal slices were prepared using a Leica VT1200s vibratome (Bannockburn, IL). Slices were prepared in ice-cold, nominally Ca^2+^-free saline containing (in mM): 125 NaCl, 2.5 KCl, 3.8 MgCl_2_, 1.25 NaH_2_PO_4_, 26.0 NaHCO_3_, 25 glucose, and 1.3 ascorbic acid, bubbled with 5% CO_2_-95% O_2_, pH 7.4. Subsequently, slices were incubated for 45 minutes at 35°C in standard ACSF containing (in mM): 125 NaCl, 2.5 KCl, 2.5 CaCl_2_, 1.3 MgCl_2_, 1.25 NaH_2_PO_4_, 26.0 NaHCO_3_, 25 glucose, and 0.1 Trolox, bubbled with 5% CO_2_-95% O_2_, pH 7.4, 315 mOsm. Slices were then incubated 4–6 hours at room temperature in either standard ACSF or modified ACSF to manipulate neuronal action potentials.

### Long-term Manipulation of Neuronal Firing Rates

To block action potential (AP)-driven synaptic transmission, slices were incubated 4–6 hours in 3.5 mM [K^+^]_o_ ACSF +1 µM of the Na^+^ channel blocker tetrodotoxin (TTX). 3.5 mM [K^+^]_o_ ACSF (317 mOsm) was used as standard ACSF (fig. 1). To increase AP-driven neurotransmitter release, 5.0 mM [K^+^]_o_ ACSF was used (320 mOsm), while 2.5 mM [K^+^]_o_ ACSF was chosen as the control condition. In 2.5 mM [K^+^]_o_, neurons fired APs very infrequently compared to 3.5 mM [K^+^]_o_ and 5.0 mM [K^+^]_o_ (fig. 1).

### Whole-cell Recording of CA3 Neuron Action Potentials

Neuronal recordings were performed as described previously (Fiacco et al. 2007). Pipettes were pulled from borosilicate glass on a Sutter P-97 Flaming/Brown type horizontal micropipette puller and not fire-polished. Pipettes had resistances of 4.6–6 MΩ when filled with a solution containing the following (in mM): 145 K-gluconate, 2 MgCl_2_, 10 HEPES, 4 Mg-ATP, 14 phosphocreatine, and 0.2 Na-GTP, pH 7.3 with KOH, 291 mOsm. Slices were incubated 4–6 hr. in either 2.5, 3.5, or 5.0 mM [K^+^]_o_ ACSF prior to recording in room temperature ACSF of the same composition. Whole-cell patch-clamp was performed using a Multiclamp 700B amplifier and PCLAMP 10.2.014 software (Molecular Devices, Sunnyvale, CA). CA3 neurons on average displayed a resting V_m_ of −61.7±1.6 mV in 2.5 mM [K^+^]_o_ ACSF (n = 12), −60.4±1.6 mV in 3.5 mM [K^+^]_o_ ACSF (n = 8) and −50.51±1.68 mV in 5.0 mM [K^+^]_o_ ACSF (n = 15). Upon attaining the whole-cell configuration, the cell membrane potential, input resistance, and access resistance were recorded. Change in any of these parameters by 20% or more was used as criteria to reject the data from further analysis. Frequency of spontaneous APs in each recording was analyzed using PCLAMP 10.2.014 software.

### Bulk-loading and Bolus-loading of Astrocytes with Ca^2+^ Indicator

Bulk-loading of astrocytes with Ca^2+^ indicator was performed as described previously [17], [18]. For standard bulk-loading experiments, 10 µM Calcium Green-1 AM and 0.076% pluronic acid in dimethyl sulfoxide (DMSO, final concentration: 0.48%) were included in the ACSF during the first 45 minute incubation period. Calcium Green-1 AM was primarily sequestered by astrocytes as observed previously for cell permeable indicators in slices from rats [1] and mice [17]. For bolus-loading experiments, 1 µM sulforhodamine 101 (SR-101) was used in place of Calcium Green-1 AM during the first 45 minute incubation period to label astrocytes. A field of astrocytes in stratum radiatum was then loaded with Oregon Green BAPTA-1 (OGB-1) AM Ca^2+^ indicator dye using a backpressure, bolus-loading technique [19]–[21]. With this technique, astrocytes 40–75 µm deep in the slice were routinely loaded and selected for subsequent imaging experiments.

**Figure 2:**
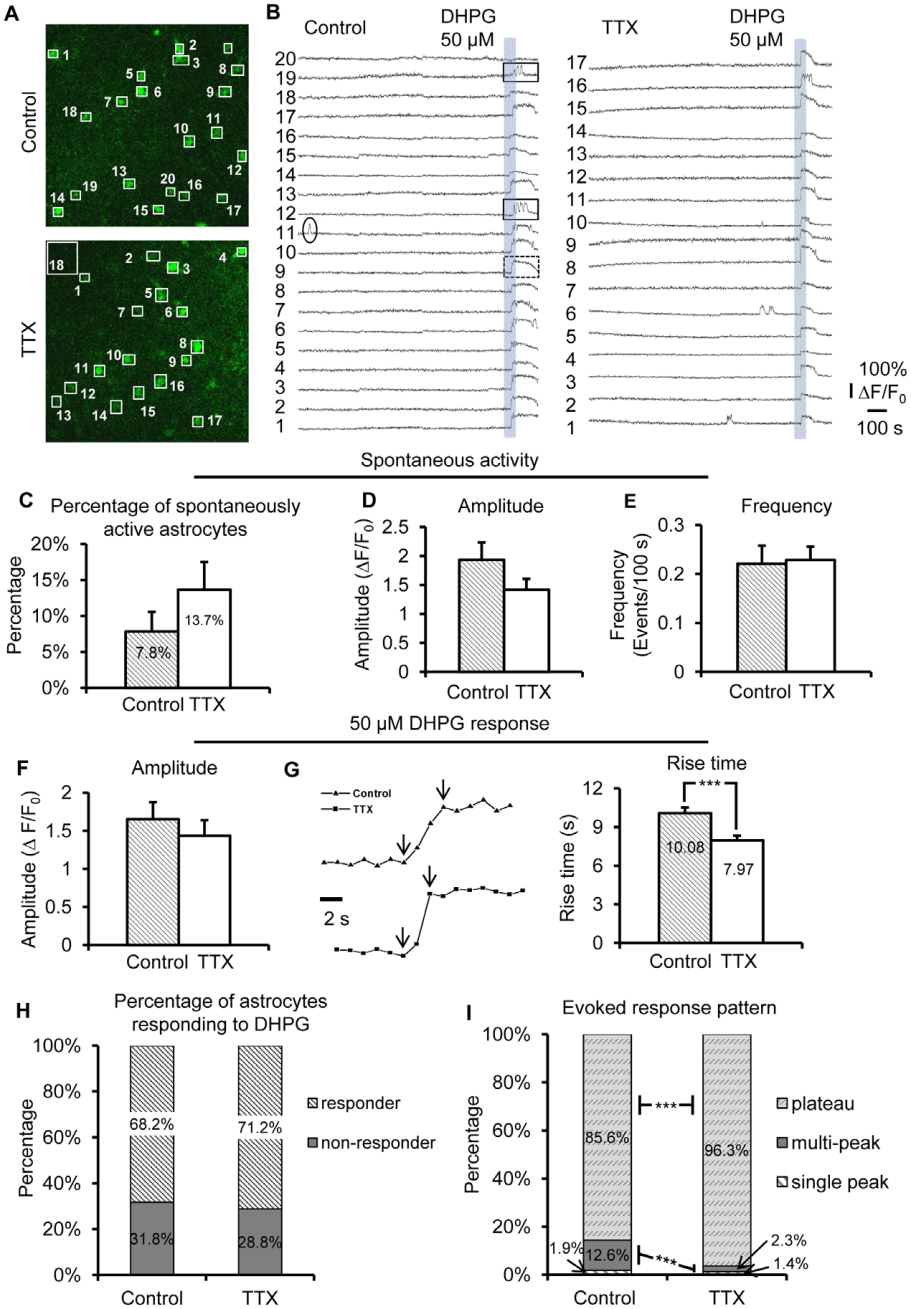
Long-term blockade of neuronal action potentials potentiates Ca^2+^ signaling in superficial bulk-loaded astrocytes. (A) CA1 s.r. astrocytes loaded with Ca^2+^ Green 1-AM. Numbered ROIs were placed over astrocyte cell bodies, and match corresponding fluorescence traces in B. Scale bar, 10 µm. (B) Representative traces of Ca^2+^ activity from each astrocyte over time. 50 µM DHPG was applied at the end of each recording to evoke group I mGluR-mediated astrocyte Ca^2+^ responses. Three different Gq GPCR Ca^2+^ elevation patterns are shown: A spontaneous Ca^2+^ transient and “single-peak” event (ellipse); agonist-evoked “multi-peak” Ca^2+^ elevations (solid rectangles); and an agonist-evoked “plateau” Ca^2+^ elevation (dashed rectangle). (C) There was a trend toward a larger percentage of astrocytes in the population exhibiting spontaneous Gq GPCR Ca^2+^ activity after 4–6 hr. incubation in TTX compared to controls. This trend became significant in subsequent bolus-loading and patch clamp experiments. (D) and (E) No difference was found in amplitude (p = 0.08) or frequency of spontaneous Ca^2+^ transients between control and TTX-treated astrocytes (24/215 astrocytes in control conditions exhibited spontaneous Ca^2+^ transients; 36/218 astrocytes in TTX exhibited spontaneous Ca^2+^ transients). (F) No change was found in amplitude of DHPG-evoked Ca^2+^ responses between control and TTX-treated astrocytes. (G) TTX-treated astrocytes had significantly faster rise times of their DHPG-evoked Ca^2+^ responses. Representative traces show the difference in rise time with arrows indicating onset and peak. (H) Overall there was no difference in the percentage of TTX-treated astrocytes responding to 50 µM DHPG with Ca^2+^ elevations compared to control. (I) Astrocytes incubated in TTX displayed a significant shift toward multi-peak and plateau type DHPG-evoked Ca^2+^ responses compared to astrocytes in control conditions (p<0.001).

### Astrocyte Patch Clamp and Loading with Ca^2+^ Indicator

Filling of astrocytes with cell-impermeant Ca^2+^ indicator via whole-cell voltage clamp was performed as described previously [22], [23]. Pipettes were pulled from borosilicate glass on a Narishige (Tokyo, Japan) PP-10 two-stage vertical pipette puller and not fire-polished. Pipettes were filled with the following internal solution (in mM): 130 K-gluconate, 4 MgCl_2_, 10 HEPES, 10 glucose, 1.185 Mg-ATP, 10.55 phosphocreatine, and 0.1315 mg/ml creatine phosphokinase, pH 7.3 by KOH, and had resistances of 7–8 MΩ. Also included in the pipette solution was 200 µM of the cell-impermeant version of OGB-1 (Invitrogen). The osmolality of this solution was 300 mOsm. Upon attaining the whole-cell configuration, the resting membrane potential of the astrocyte was recorded in current clamp mode. Astrocytes displayed a resting V_m_ of −77.8±1.2 mV in 2.5 mM [K^+^]_o_ ACSF, −73.5±0.7 mV in 3.5 mM [K^+^]_o_ ACSF and −69.7±1.5 mV in 5.0 mM [K^+^]_o_ ACSF, with average input resistances of <10 MΩ. Then astrocytes were voltage-clamped at a holding potential of −90 mV from which a voltage-step protocol was initiated to verify the passive electrical properties of the astrocyte. A test pulse of −5 mV was included after each voltage step in order to monitor changes in access resistance. Astrocyte current signals were low-pass filtered at 2 kHz and digitized at 100 kHz via a Digidata 1440 (Molecular Devices, Sunnyvale, CA). Astrocytes were voltage-clamped for no longer than 3 min., at which time the patch pipette was gently retracted from the cell. A smooth, stable off-cell and formation of an outside-out patch was a strong indicator of minimal damage to the cell membrane during the patch clamp procedure. Then the astrocytes were allowed to rest for 10 min. prior to initiation of image acquisition. In some experiments, astrocytes were patch clamped a second time with 200 µM Alexa Fluor 568 hydrazide dye (Invitrogen) to determine the amount of the astrocyte filled by OGB-1.

### Confocal Imaging and Recording of Astrocyte Ca^2+^ Activity

An Olympus Fluoview 1000 confocal microscope, software, and LUMPlanFl 60×/1.10NA water immersion objective (Center Valley, PA) were used to record astrocytic Ca^2+^ activity. The green Ca^2+^ indicators (Ca^2+^ Green 488 1-AM; OGB-1 AM, or OGB-1 hexapotassium salt) were excited using a 488 nm argon laser, and emission was collected using a 503–548 bandpass filter. Single plane, 512×512 pixel x-y-t scans of 2.19 µm optical section thickness were used to record astrocyte Ca^2+^ activity. The pixel size was 12-bit, 0.276 µm/pixel. The scan interval was 1.2 s and data were collected over the course of 40 min. Individual astrocytes in CA1 stratum radiatum were identified first by their location, size, and morphology. In bulk-loaded slices astrocytes were further identified by their sequestering of Ca^2+^ indicator and characteristic long-duration Ca^2+^ responses compared to neurons [24]. In bolus-loading experiments, overlay of the SR-101 and OGB-1 signals was used to identify bolus-loaded cells as astrocytes [21]. All recordings (including controls) were performed in 1 µM TTX to eliminate potential neuronally-evoked astrocytic Ca^2+^ responses. Recordings were completed within 40 minutes to limit any possible TTX-induced scaling effects on control slices. All imaging was performed using 0.5% laser power to limit the possibility of photosensitivity induced spontaneous Ca^2+^ oscillations [2]. Only a single (field of) astrocyte(s) was recorded per slice to eliminate the possibility of double-sampling or changes due to multiple agonist applications. Astrocytes exhibiting at least one spontaneous Ca^2+^ elevation during 10 to 15 minutes baseline recording were counted as spontaneously active cells.

**Figure 3:**
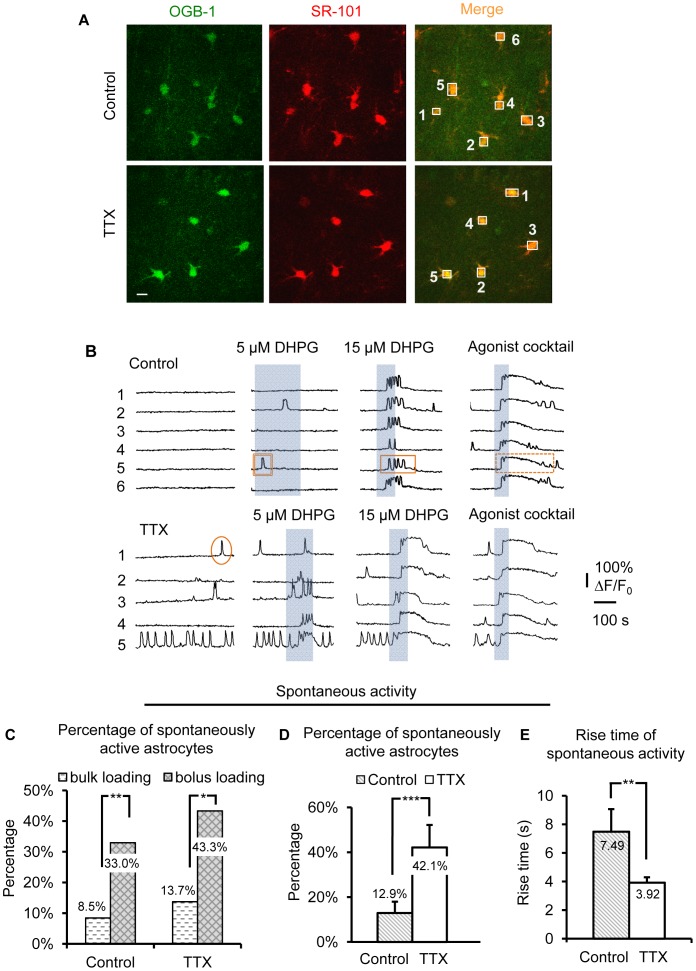
Astrocyte spontaneous Gq GPCR activity is enhanced after 4–6 hr incubation in TTX. (A) SR-101 and OGB-1 AM loaded astrocytes in a control and TTX-incubated slice. Numbered ROIs match Ca^2+^ traces in (B). Scale bar, 10 µm. (B) Representative Ca^2+^ activity over time in astrocyte cell bodies. Ellipse indicates spontaneous Ca^2+^ transient; double rectangle, single rectangle with solid outline and dashed rectangle indicate agonist-evoked single peak, multi-peak, and plateau type Ca^2+^ elevations, respectively. Summary of DHPG-evoked responses is presented in figure 4. Note that lower concentrations of DHPG were applied for a longer period of time, especially in the control condition, as it was difficult to determine if the cells were responding to the agonist. TTX-treated astrocytes gave much more obvious responses to 5 µM DHPG compared to control. (C) The percentage of spontaneously active astrocytes in deeper tissue was significantly higher than astrocytes loaded near the slice surface (Control bolus-loaded: n = 40; Control bulk-loaded: n = 215; TTX bolus-loaded: n = 30; TTX bulk-loaded: n = 218). (D) The percentage of astrocytes exhibiting spontaneous Ca^2+^ transients in the soma and main processes was significantly increased following TTX treatment compared to control. (E) Rise time of the spontaneous Ca^2+^ transients was significantly faster in TTX-treated astrocytes (n = 27) compared to controls (n = 32).

**Figure 4:**
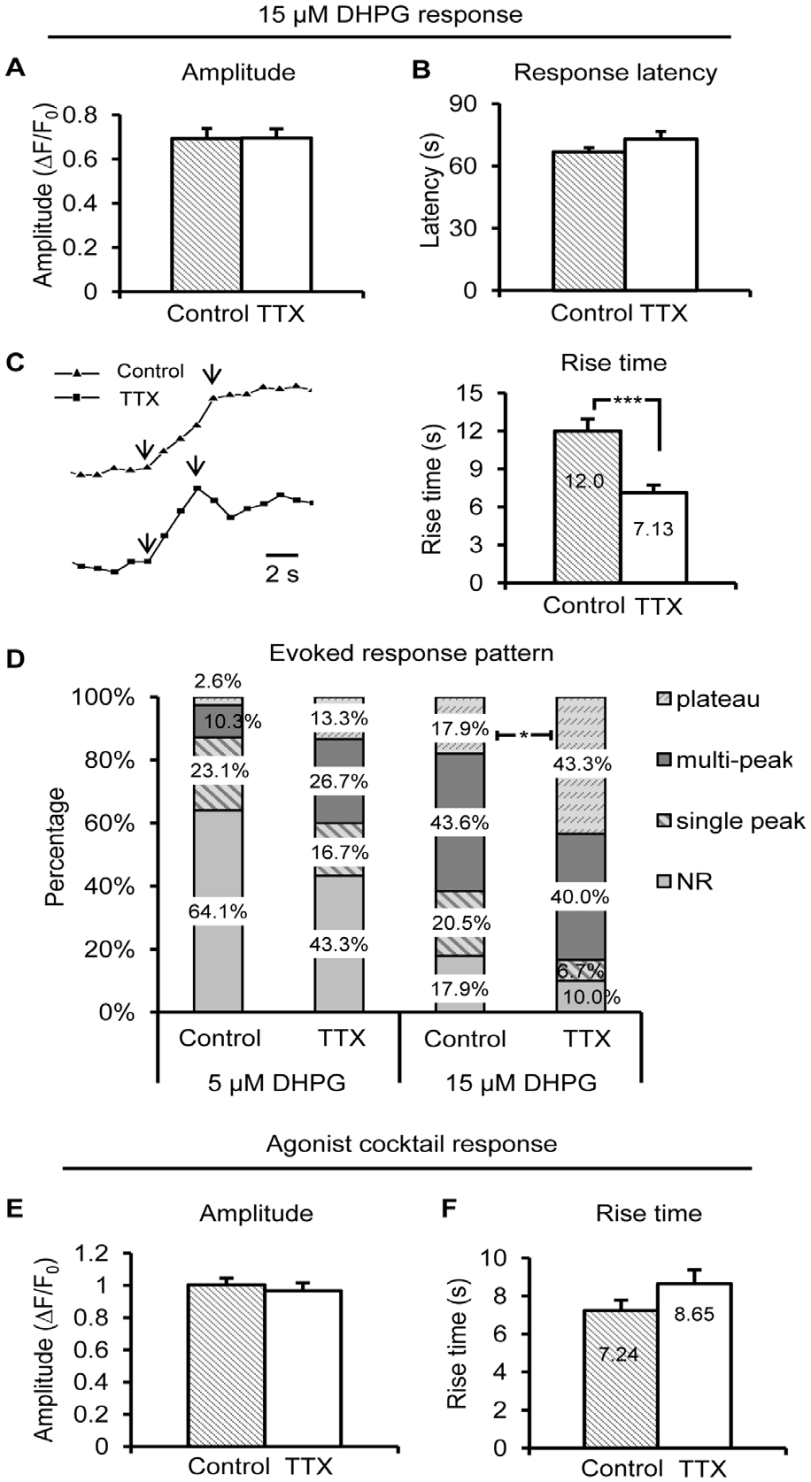
Astrocyte evoked Group I mGluR activity is enhanced after 4–6 hr incubation in TTX. (A) and (B) The amplitude and response latency of evoked Ca^2+^ responses to 15 µM DHPG were unchanged after 4–6 hr. incubation in TTX compared to control. (C) Rise time of the evoked Ca^2+^ responses to 15 µM DHPG was significantly faster in TTX treated astrocytes (n = 27) compared to controls (n = 32). (D) A greater percentage of TTX-treated astrocytes responded to low concentrations of DHPG, with a shift toward a plateau pattern compared to control astrocytes. Please refer to representative traces in Figure 3B. (E) and (F) The amplitude and rise time of Ca^2+^ responses to agonist cocktail were unchanged.

**Figure 5:**
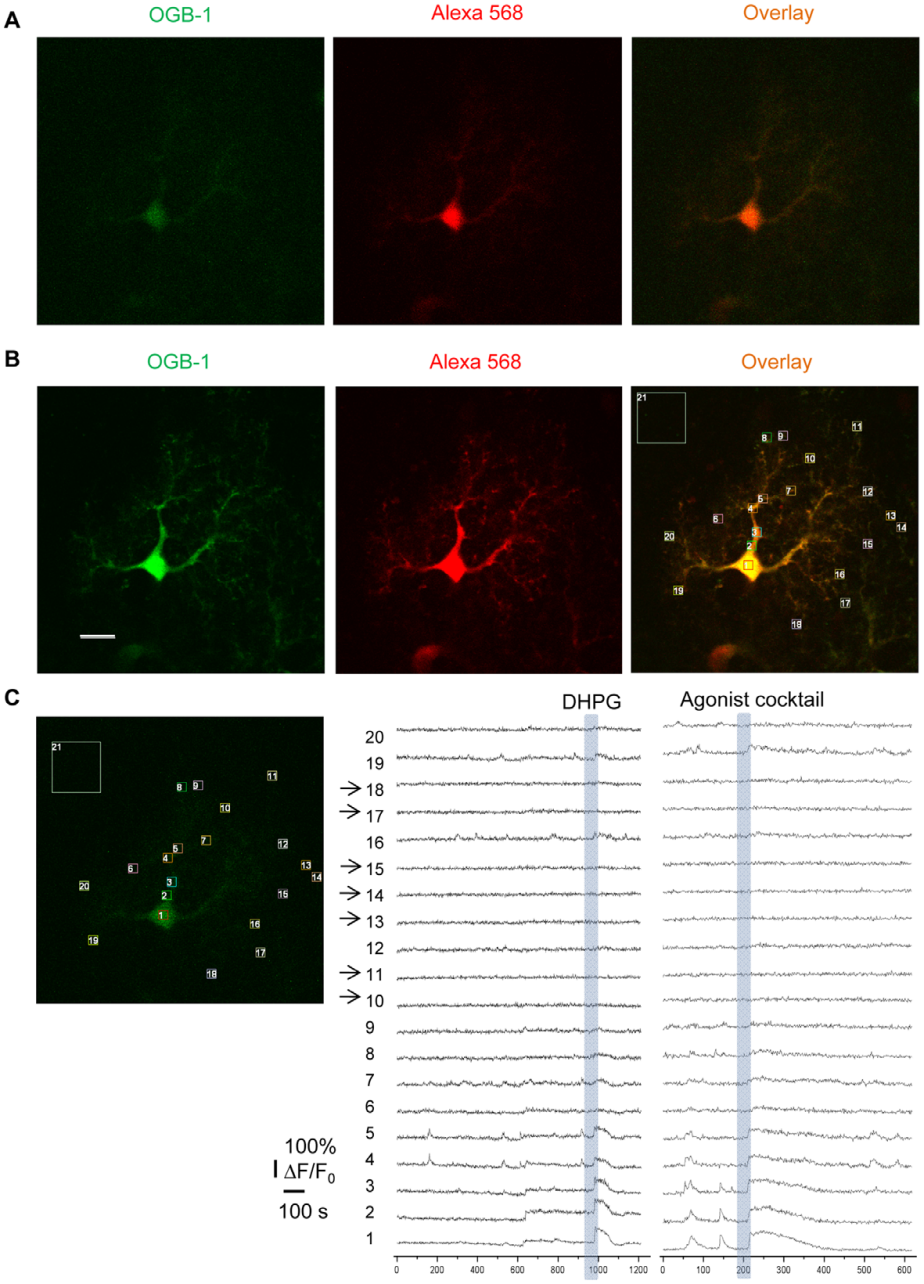
Patch clamp of astrocytes with OGB-1 Ca^2+^ indicator dye enables recording of Ca^2+^ transients in fine astrocyte processes. (A) Single astrocyte filled with OGB-1 and Alexa 568 via patch pipette. Images were obtained using the same laser power with which Ca^2+^ recording was performed. (B) The same astrocyte as in (A) imaged using higher laser power reveals the fine processes after Ca^2+^ recording. ROIs were placed over the fine processes of the astrocyte using the Alexa 568 overlay. Scale bar, 10 µm (images shown at same magnification as those in (A)). (C) Calcium activity over time within ROIs indicated in (B). Traces without arrows indicate spontaneous and agonist-evoked Ca^2+^ activity in the astrocyte. Arrows indicate ROIs that were placed where there was no visible Alexa 568 signal (background).

**Figure 6:**
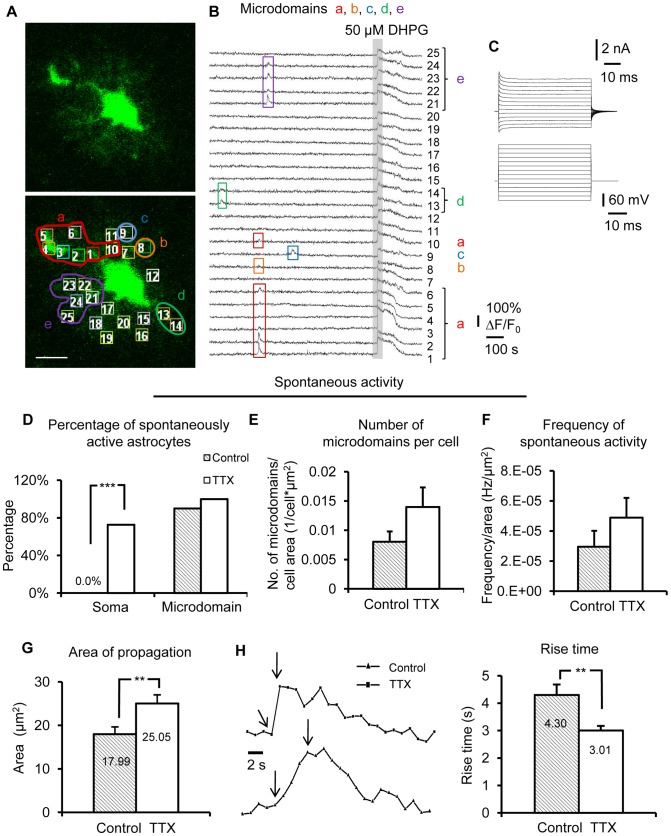
Long-term blockade of neuronal APs potentiates spontaneous Ca^2+^ signaling in astrocyte microdomains. (A) OGB-1 loaded single astrocyte with equal sized boxes placed over its fine processes, which are visible in the left panel. Scale bar, 10 µm. (B) Traces of spontaneous microdomain Ca^2+^ activity and DHPG-evoked Ca^2+^ responses match numbered ROIs from cell in (A). (C) The astrocyte in (A) exhibited passive currents when the membrane was stepped from −180 to +80 mV in 20 mV increments. A test pulse of −5 mV was included after each voltage step in order to monitor changes in access resistance. (D) After TTX treatment, spontaneous Ca^2+^ oscillations frequently occurred in the astrocyte soma (n = 11), while most astrocytes in control conditions very rarely had spontaneous somatic Ca^2+^ transients (n = 10). (E) and (F) TTX treated astrocytes (n = 58 microdomains/11 cells) exhibited a trend toward a greater number of microdomains per cell compared to controls (n = 29 microdomains/10 cells). There was no change in the frequency of spontaneous microdomain Ca^2+^ activity. (G) and (H) Spontaneous microdomain Ca^2+^ transients had significantly larger areas of propagation and a faster rise time after TTX treatment.

**Figure 7:**
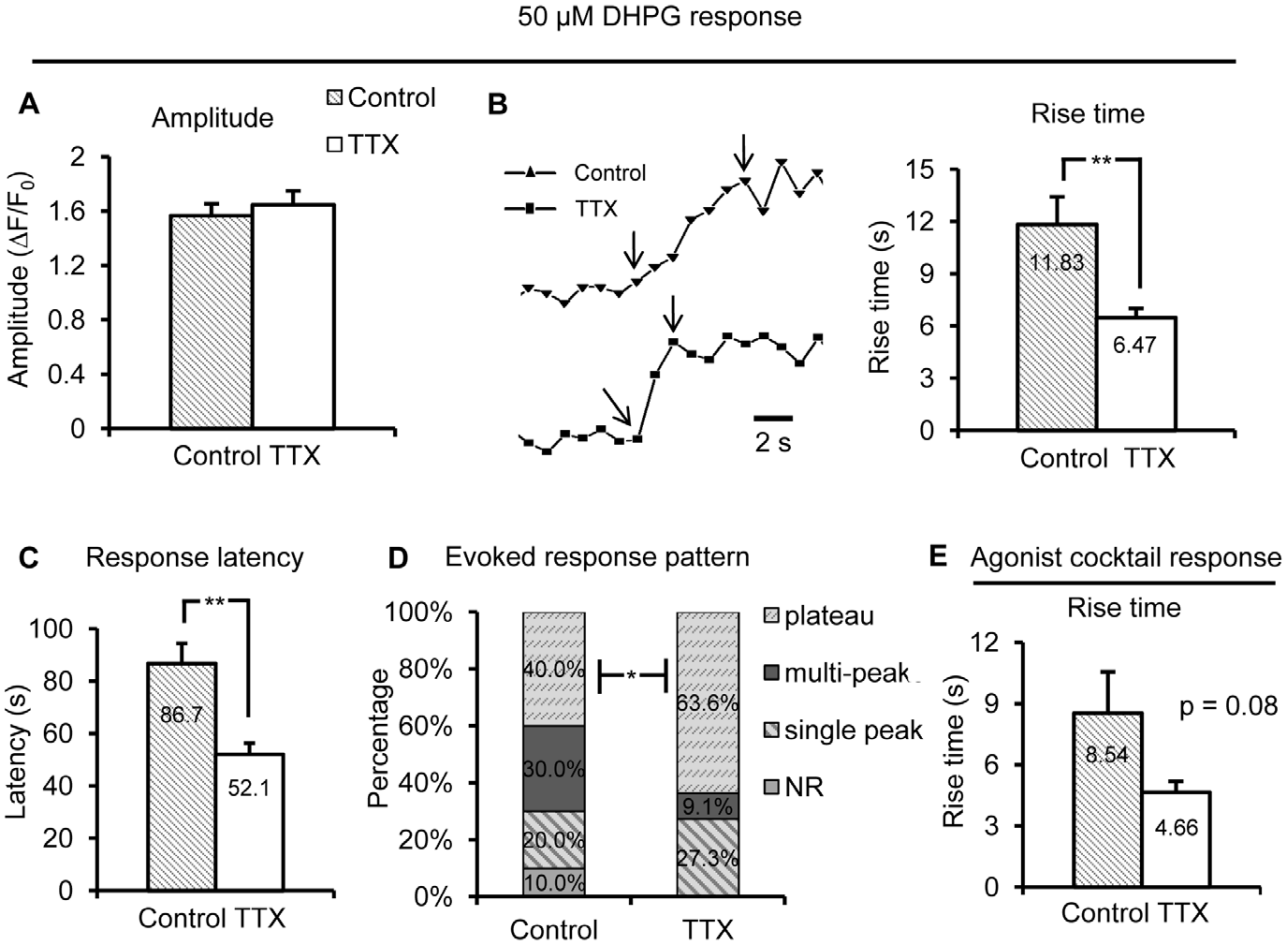
Long-term blockade of neuronal APs potentiates evoked group I mGluR Ca^2+^ responses in astrocyte microdomains. (A), (B) and (C) While amplitude remained unchanged, Ca^2+^ elevations in TTX-treated astrocytes (n = 56 microdomains/11 cells) exhibited a faster rise time and a shorter latency to respond to 50 µM DHPG compared to controls (n = 28 microdomains/10 cells). (D) The pattern of astrocyte microdomain Ca^2+^ responses evoked by 50 µM DHPG shifted from the weaker single peak phenotype towards the more robust plateau type after TTX treatment. (E) Rise time of astrocyte microdomain Ca^2+^ responses evoked by agonist cocktail was not significantly different in the TTX treated *vs*. control group (control: n = 40 cells; TTX: n = 30 cells).

**Figure 8:**
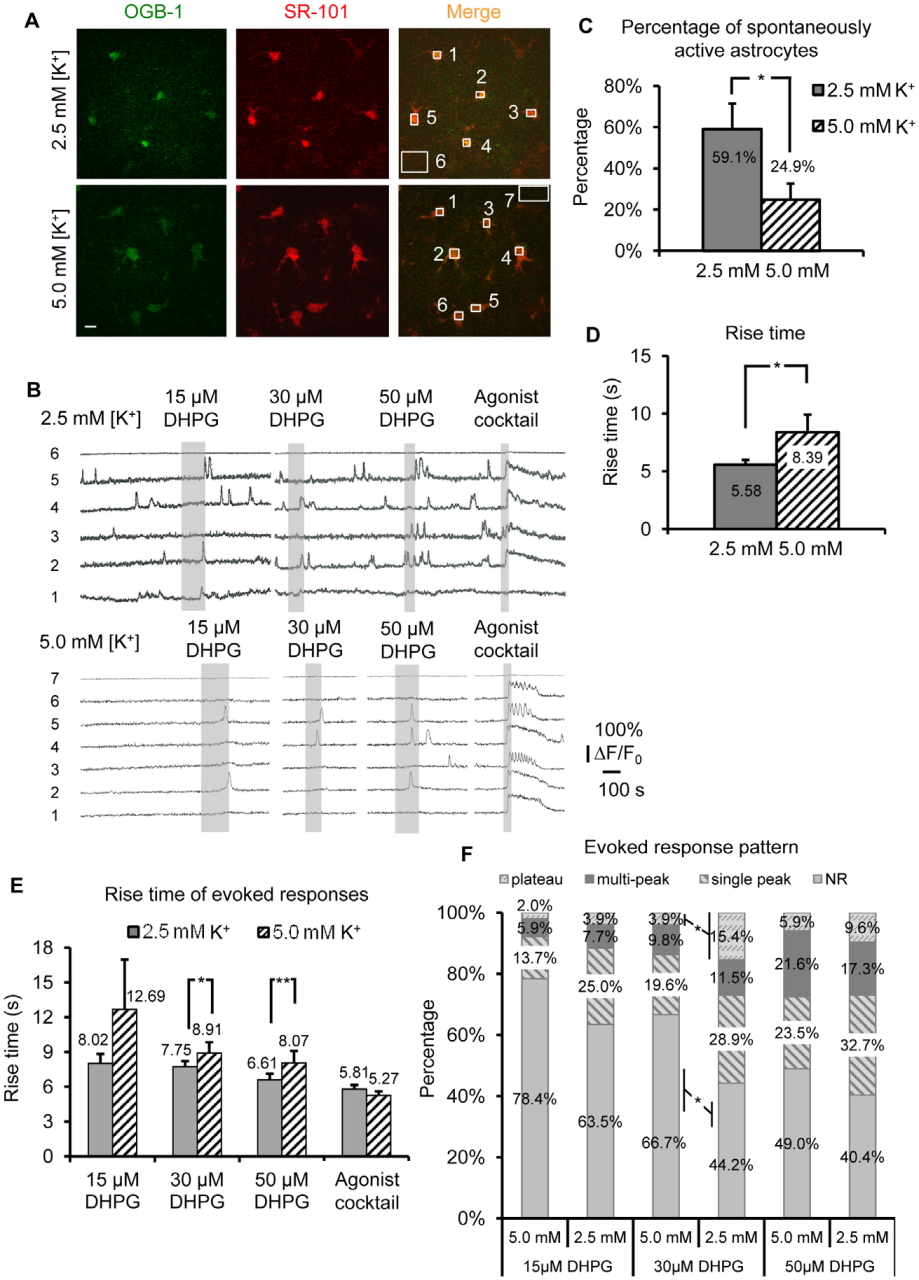
Increased neuronal firing rates depress spontaneous and evoked group I mGluR astrocyte Ca^2+^ transients. (A) SR-101 and OGB-1 loaded astrocytes in 2.5 mM and 5.0 mM [K^+^]_o_ incubated slices. Numbered ROIs match Ca^2+^ traces in (B). Scale bar, 10 µm. (B) Calcium activity from 2.5 mM and 5.0 mM [K^+^]_o_ incubated slices shown in (A). 5.0 mM [K^+^]_o_ treated astrocytes exhibited fewer spontaneous somatic Ca^2+^ transients and weaker DHPG evoked responses compared to 2.5 mM [K^+^]_o_ ACSF. Note that DHPG application times tended to be longer in 5.0 mM [K^+^]_o_ treated astrocytes compared to control, as it required more time before a Ca^2+^ response was clearly evident in experimental *vs*. control conditions. Also, cell 1 in 2.5 mM [K^+^]_o_ ACSF was rejected from further analysis as it did not respond to agonist cocktail. (C) The percentage of spontaneously active astrocytes was significantly lower in 5.0 mM [K^+^]_o_ treated (n = 51 cells/10 slices) *vs*. control slices (n = 52 cells/10 slices). (D) Rise time of somatic spontaneous Ca^2+^ transients in 5.0 mM [K^+^]_o_ treated astrocytes (n = 13) was significantly slower compared to controls (n = 30). (E) Rise time of evoked somatic astrocyte Ca^2+^ responses to three concentrations of DHPG was slower in 5.0 mM [K^+^]_o_ treated *vs*. control astrocytes. (F) At all DHPG concentrations tested, a lower percentage of 5.0 mM [K^+^]_o_ treated astrocytes responded to DHPG, and the pattern of responses shifted toward the weaker single-peak phenotype, compared to 2.5 mM [K^+^]_o_ incubated astrocytes.

**Figure 9:**
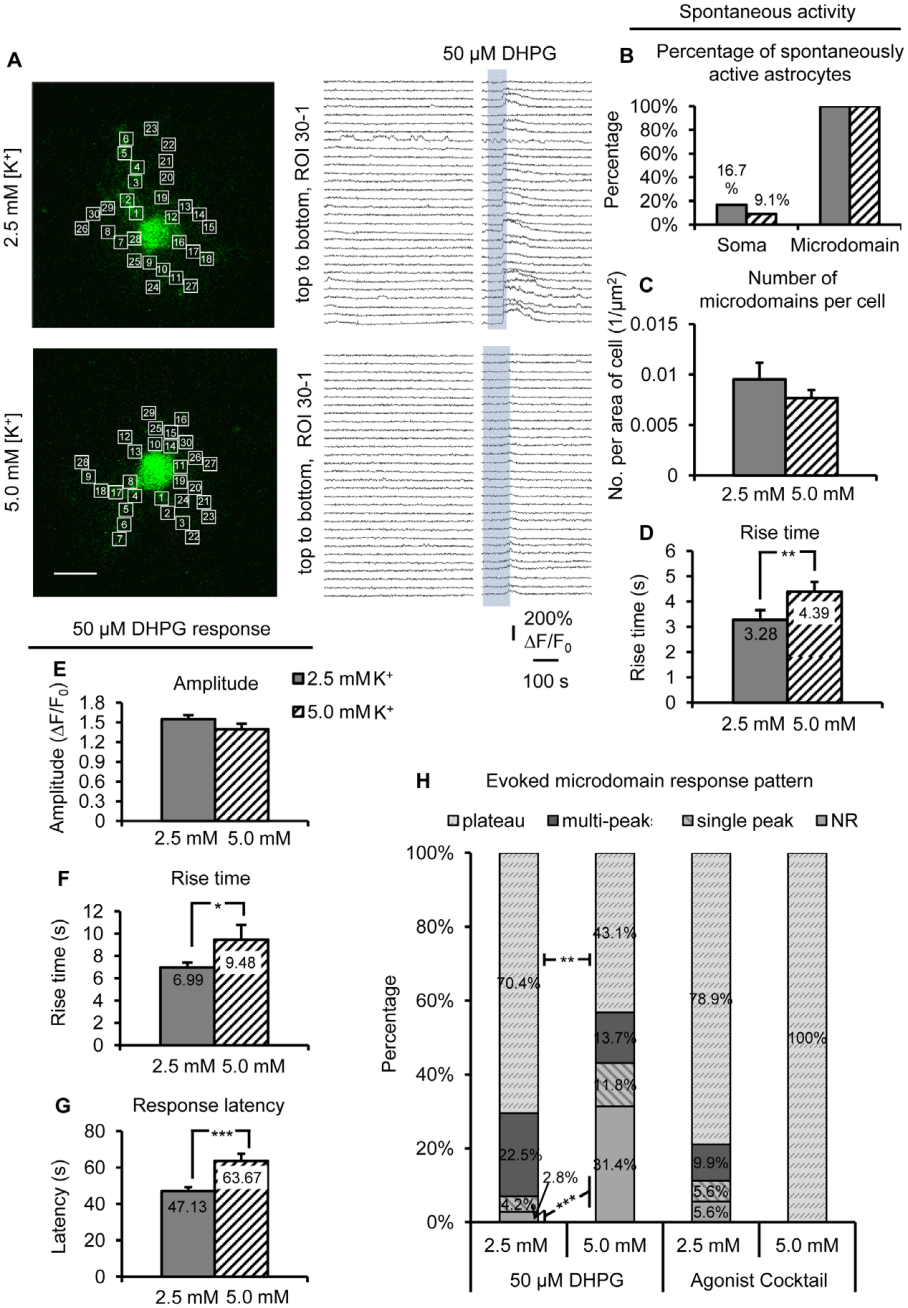
Increased neuronal firing rates depress spontaneous and evoked group I mGluR Ca^2+^ transients in microdomains. (A) OGB-1 loaded astrocytes treated with either 2.5 mM or 5.0 mM [K^+^] ACSF. Cells are shown with equal sized (3.5 µm/side) ROIs placed over astrocyte processes. Traces of spontaneous microdomain Ca^2+^ activity and 50 µM DHPG-evoked Ca^2+^ elevations match numbered ROIs in the images. (B) Spontaneous Ca^2+^ transients occurred in microdomains of all s.r. astrocytes, while a lower percentage of 5.0 mM [K^+^]_o_ treated astrocytes (n = 14) had spontaneous Ca^2+^ activity in the soma compared to control astrocytes (n = 12). (C) 5.0 mM [K^+^]_o_ treated astrocytes exhibited a trend toward a lower number of spontaneous Ca^2+^ microdomains per cell compared to controls (n = 51 microdomains/11 cells in 5 mM [K^+^]_o_; n = 71 microdomains/12 cells in 2.5 mM [K^+^]_o_). (D) Rise time of spontaneous microdomain Ca^2+^ transients was slower after 4–6 hr. incubation in 5.0 mM [K^+^]_o_ compared to control [K^+^]_o_. (E), (F) and (G) Ca^2+^ elevations evoked by DHPG in 5.0 mM [K^+^]_o_ treated astrocytes (n = 37 microdomains/11 cells) had a significantly slower rise time and longer response latency compared to those in the control condition (n = 69 microdomains/12 cells). Amplitude of microdomain DHPG responses was unchanged. (H) Summary of the evoked Ca^2+^ response patterns in astrocyte microdomains. 31.4% of microdomains in 5.0 mM [K^+^]_o_ treated astrocytes did not respond to 50 µM DHPG, while only 2.8% of microdomains in 2.5 mM [K^+^]_o_ treated astrocytes did not respond to the agonist. There was also a significant shift away from the plateau responses in the microdomains after incubation in 5.0 mM [K^+^]_o_ toward the weaker, single-peak response type.

**Figure 10:**
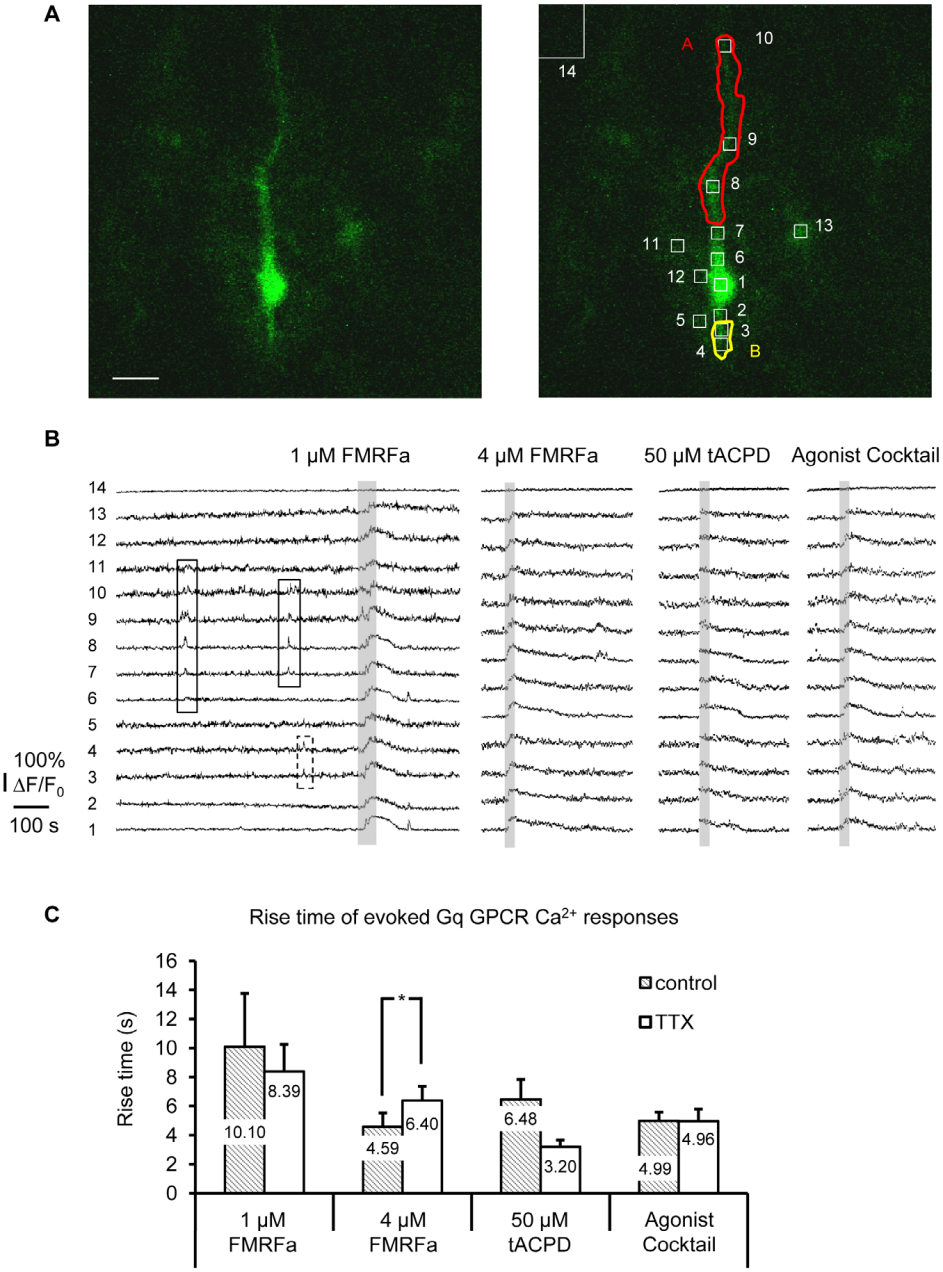
Astrocytic MrgA1R Ca^2+^ signaling is not affected by long-term blockade of neuronal APs. (A) A single MrgA1R^+^ hippocampal s.r. astrocyte filled with OGB-1 Ca^2+^ indicator dye by patch clamp from a control-incubated hippocampal slice. Equal sized boxes of 3.5 µm/side (12.3 µm^2^) were placed over astrocyte processes to study microdomain Gq GPCR activity (top middle panel) and match corresponding Ca^2+^ traces in (B). Scale bar, 10 µm. (B) Representative traces of Ca^2+^ activity from all ROIs in the same astrocyte shown in (A). The Ca^2+^ trace at the bottom is from the cell soma, which is ROI #1. The MrgA1R agonist FMRFa evoked Ca^2+^ elevations similar to those produced by tACPD and agonist cocktail. The spontaneous Ca^2+^ activity in microdomains A and B are highlighted by solid and dashed rectangles, respectively. (C) The rise time of Ca^2+^ responses evoked by 1 and 4 µM FMRFa was no different in TTX treated *vs*. control astrocytes (control: n = 9 microdomains/8 cells; TTX: n = 14 microdomains/10 cells). The rise time of tACPD evoked Ca^2+^ responses was significantly faster in TTX treated astrocytes, similar to observations using DHPG in wild type hippocampal slices.

### Agonist Application and Monitoring of Agonist-evoked Astrocyte Ca^2+^ Elevations

Drugs were bath-applied using an electronic valve controller (Warner Instrument, Hamden CT). The group I mGluR agonist (RS)-3,5-DHPG (at 5, 15, 30, or 50 µM) or the group I and II agonist trans-ACPD (50 µM) was applied to evaluate evoked group I mGluR Ca^2+^ responses in astrocytes. Agonist was applied not for a specific duration, but until a clear response was evident. The reason for this was twofold: First, often different agonist concentrations were used. Lower agonist concentrations require longer wash-in times to reach sufficient tissue concentration to bind a sufficient number of receptors to evoke a response. Second, the experimental manipulations (TTX or elevated [K^+^]_o_) clearly affected response latency to a fixed concentration of agonist, presumably because the functional expression of the receptors had changed. All recordings were performed in 1 µM TTX to eliminate neuronally-driven astrocyte Ca^2+^ elevations that may result from agonist activation of neuronal group I mGluRs [25], [26]. A minimum of 5 min recording was included after Ca^2+^ returned to baseline in-between DHPG applications to limit receptor desensitization of group I mGluRs. A Gq GPCR agonist cocktail consisting of 10 µM each of histamine, carbachol, and ATP to stimulate astrocytic histamine H1, muscarinic acetylcholine, and purinergic receptors, respectively, was applied for two main purposes: 1) as a positive control for viable astrocytes that did not respond to DHPG; and 2) to determine the specificity of changes to group I mGluRs *vs*. other astrocytic Gq GPCRs. Cells which failed to respond to agonist cocktail were excluded from subsequent analysis.

### Analysis of Astrocyte Gq GPCR Ca^2+^ Activity and Statistics

Slices or single astrocytes from bolus-loading and patch clamp experiments were given a numeric code and analyzed blindly. Square-shaped regions of interest (ROIs) sized to match the cell soma (for bulk-loading and bolus-loading experiments) or fixed at 3.5 µm/side (for patch-clamp experiments for analysis of astrocytic subcompartments) were placed over the astrocyte soma and/or processes using Olympus Fluoview 1000 software. Increases in average fluorescence intensity normalized to baseline fluorescence (ΔF/F_0_) within each ROI represented increases in Ca^2+^ concentration [27]. Such increases in ΔF/F_0_ values were scored as Ca^2+^ elevations if the peak amplitude was greater than two standard deviations above the mean of baseline fluorescence for at least two sample points. For patch clamp experiments, within each astrocyte, microdomains were identified as compartments of astrocyte processes that exhibited spontaneous Ca^2+^ elevations independently from other parts of the cell. It is well-documented that the smallest astrocyte processes are too small to be resolved by confocal or 2-photon microscopy [28]. However, Ca^2+^ activity was clearly resolved in ROIs over “bulk” fine processes that appeared as a cloud-like fuzz at the astrocyte periphery. Microdomains occurring at the same time were scored as separate events if intervening ROIs did not exhibit Ca^2+^ elevations. Within each microdomain, the ROI that corresponded to the “initiation site” of the spontaneous or evoked Ca^2+^ transient was identified based on the earliest onset among all ROIs in the same microdomain, and was later used to represent this microdomain for further analysis. Spontaneous Ca^2+^ transients covering ≥75% of the planar area of the astrocyte were defined as “whole cell” Ca^2+^ elevations and analyzed separately.

The amplitude, frequency, and kinetics of spontaneous Gq GPCR signaling events and agonist-evoked mGluR responses were determined for each microdomain. The average baseline fluorescence intensity was calculated by averaging 30 seconds of stable baseline immediately preceding each Ca^2+^ elevation. The onset of each Ca^2+^ elevation was defined as the last data point before the fluorescence intensity went one standard deviation above the mean, and the peak of the Ca^2+^ elevation was defined as the first peak fluorescence intensity (in cases of “multipeak” responses the first peak was used). Because the frequency of spontaneous Ca^2+^ transients observed in astrocyte processes may be affected by the area of each astrocyte that can be imaged, frequency was normalized to the astrocyte area prior to comparison between groups. Rise time was defined as the difference between the response onset and the time corresponding to the peak amplitude (0 to 100%). For agonist-evoked responses, latency was calculated as the time between initiation of agonist perfusion to the peak of the response. Analysis of rise time was chosen in addition to response latency due to long wash-in time of agonists in hippocampal slices *vs*. cultured cells, which might obscure potential significant differences in response latency.

For agonist-evoked Ca^2+^ responses, specific response patterns were grouped into 3 categories: “single peak”, “multipeak”, or “plateau”. Single peak responses were defined as a response with only one data point within ±10% of the peak value. Plateau responses maintained the peak amplitude for at least 3.6 seconds, and usually for much longer. Therefore, the duration of single peak responses was much shorter compared to plateau Ca^2+^ responses. Multi-peak responses were defined as a minimum of at least 2 single peaks in rapid succession, with ≤10.8 s gap between each single peak. In addition, multipeak responses were defined only when the fluorescence intensity fell below 50% amplitude after the first peak prior to rising again for secondary peaks. If the second peak occurred before the first peak decayed by at least 50%, this event was defined as a plateau response. Cells or microdomains that did not produce Ca^2+^ elevations to group I mGluR agonist were grouped as non-responders (NR) as long as these cells were deemed viable based on responses to agonist cocktail. Analysis of agonist-evoked response duration and area was performed only for plateau responses.

Analyses were performed using number of astrocytes as ‘n’ for bulk-loading and bolus loading experiments, and number of microdomains for patch-clamp experiments. Error bars represent S.E.M. in all figures. Student’s independent *t*-test was used for statistical comparison of means between the control and treated groups. Pearson’s chi-square test was used for comparison of the Ca^2+^ activity patterns between control and treatment groups. Fisher’s exact 2-tail test was used for comparison of percentages of specific Ca^2+^ activity patterns between control and treatment groups. Significance is described at the *0.05, **0.01, or ***0.001 levels.

## Results

### Firing Rates of CA3 Neurons in Hippocampal Slices as a Function of [K^+^]_o_

As a first step to determine if astrocytic Gq GPCR activity is sensitive to changes in neuronal action potentials (APs), we measured the effect of different extracellular K^+^ concentrations ([K^+^]_o_) on AP frequency of CA3 pyramidal neurons (fig. 1). CA3 neurons in 2.5 mM [K^+^]_o_ on average rested at −61.7±1.6 mV (n = 12; fig. 1B) and only very rarely fired spontaneous APs (0.00016±8.9*10^−5^ Hz; n = 12; fig. 1A, C and D). In 3.5 mM [K^+^]_o_, CA3 neurons were more depolarized (n = 8; fig. 1B) and fired more frequently (0.02±0.0088 Hz; n = 8; fig. 1C). This difference in firing frequency was not significant (p = 0.054), probably due to the large variability among individual recordings (fig. 1D). However, in 5.0 mM [K^+^]_o_, CA3 neurons exhibited a 10 mV depolarizing shift in resting membrane potential (n = 15; fig. 1B) and a significantly higher rate of spontaneous APs compared to CA3 neurons in 2.5 mM [K^+^]_o_ (n = 15; fig. 1C and D). Based on these results, 3.5 mM [K^+^]_o_ ACSF was chosen as the control condition in experiments to determine the effect of long-term block of neuronal APs in TTX, while 5.0 mM [K^+^]_o_ ACSF was chosen to determine the effect of long-term increase in neuronal APs as compared to 2.5 mM [K^+^]_o_.

### Long-term Blockade of Neuronal Action Potentials Potentiates Ca^2+^ Signaling in Superficial Bulk-loaded Astrocytes

Previous studies *in vitro* have provided evidence indicating how changes in Gq GPCR expression levels correlate to specific changes in spontaneous and agonist-evoked Ca^2+^ elevations [7]–[12], [14], [15]. Therefore, as an initial experiment to determine if astrocytes adjust their functional expression of Gq GPCRs after long-term changes in neuronal firing rates, Gq GPCR Ca^2+^ activity was recorded in the soma of stratum radiatum (s.r.) astrocytes in acute mouse hippocampal slices bulk-loaded with the Ca^2+^ indicator Calcium Green-1 AM [1], after 4–6 hr incubation in either 3.5 mM [K^+^]_o_ ACSF or 3.5 mM [K^+^]_o_ ACSF plus 1 µM TTX (fig. 2). The advantage of bulk-loading Ca^2+^ indicator is that spontaneous and evoked Gq GPCR activity can be recorded from a large population of astrocytes, but with the following disadvantages: The signal-to-noise is not sufficient to record reliably from small astrocyte compartments [29], and only astrocytes near the surface of the slice take up the dye as shown in figure 2A [17]. Spontaneous Ca^2+^ transients were recorded in the soma of s.r. astrocytes (fig. 2B). Previous studies have indicated that a primary target of neuron-to-astrocyte receptor communication is astrocytic group I mGluRs [1], [2], [6]. Therefore, after the 15 min baseline recording of spontaneous Ca^2+^ activity, slices were perfused with 50 µM of the group I mGluR agonist DHPG to evoke group I mGluR-mediated astrocyte Ca^2+^ elevations (fig. 2B). Astrocyte responses to 50 µM DHPG were of two main types: multi-peak or plateau (fig. 2B). A very low percentage of astrocytes responded to 50 µM DHPG with single peak Ca^2+^ elevations (fig. 2I).

Overall, there was no effect of 4–6 hr incubation in TTX on the percentage of astrocytes in the population exhibiting spontaneous Gq GPCR activity compared to control conditions (p = 0.20; fig. 2C). Furthermore, no significant differences were found between control and TTX incubated slices in the amplitude or frequency of the spontaneous astrocytic Ca^2+^ elevations (control: n = 215 cells/15 slices; TTX: n = 218 cells/16 slices; fig. 2D and E). The percentage of astrocytes responding to 50 µM DHPG was similar between the two groups (71.2% *vs*. 68.2%) (fig. 2H). There was also no change in either the amplitude (fig. 2F) or duration (data not shown) of the DHPG-evoked responses in TTX *vs*. control incubated slices. However, the rise time of DHPG-evoked Ca^2+^ responses was significantly faster after incubating slices in TTX (p<0.001; fig. 2G). These results fit with previous reports indicating that a primary effect of changes in expression levels of Gq GPCRs are changes in agonist evoked Ca^2+^ response latency and rise time, without accompanying changes in amplitude [10]–[12], [30].

Previous studies have also revealed a dose-dependent effect of group I mGluR agonist concentration on the pattern of the Ca^2+^ response, with lower concentrations leading to single-peak or oscillating (“multipeak”) responses while higher concentrations produce long-lasting plateau responses [7], [14], [15]. This stepwise dose-response effect of increasing agonist concentration is presumably the effect of stimulating a greater number of existing receptors because the probability of agonist-receptor binding will increase at higher agonist concentrations. Therefore, we reasoned that the response pattern will shift at a fixed concentration of agonist if the expression or activity levels of the receptors has changed. As expected, in TTX-treated slices a significantly higher percentage of astrocytes elicited plateauing DHPG responses compared to controls (p<0.001), while control slices had a significantly greater percentage of multi-peak responses (p<0.001, fig. 2I). Taken together, these data suggest that Ca^2+^ signaling of astrocytic group I mGluRs increases after long-term blockade of neuron-to-astrocyte receptor signaling.

### Astrocytic Ca^2+^ Signaling is Also Potentiated Using Backpressure Loading of Astrocytes Deeper in Tissue

We had some concern about standard bulk-loading procedures to load astrocytes with Ca^2+^ indicator. Astrocytes taking up the Ca^2+^ indicator tend to be very near the slice surface, where astrocytes and their synaptic associations may be compromised during slice preparation. To address this concern we loaded s.r. astrocytes with OGB-1 AM Ca^2+^ indicator dye using a more recently developed backpressure, bolus-loading protocol [19]–[21]. By pressure-ejecting the Ca^2+^ indicator 40–75 µm into the slice, we obtained improved resolution and signal-to-noise in deeper, healthier tissue compared to conventional bulk-loading protocols. Overlay of the SR-101 and OGB-1 AM signals was used to identify bolus-loaded cells as astrocytes (fig. 3A). In general, astrocytes in TTX-treated hippocampal slices exhibited more spontaneous Ca^2+^ transients and responded to DHPG more readily compared to astrocytes in control slices (fig. 3B). Responses to a Gq GPCR agonist cocktail consisting of 10 µM each of histamine, carbachol, and ATP, however, were the same as in control, suggesting that changes in evoked astrocyte Ca^2+^ signaling were mainly due to changes in group I mGluRs.

The percentage of spontaneously active astrocytes bolus-loaded with Ca^2+^ indicator was higher than in bulk-loaded slices for both control (p<0.01; bolus-loaded: 12.9% ±5.1%; n = 40 cells/8 slices; bulk-loaded: 7.8% ±2.7%; n = 215 cells/15 slices), and TTX incubated slices (p<0.05; bolus loaded: 42.1% ±10.1%; n = 30 cells/7 slices; bulk-loaded: 13.7% ±3.8%; n = 218 cells/16 slices) (fig. 3C). The increase in astrocyte Ca^2+^ signaling activity deeper in the slice suggested that connections among astrocyte processes and neuronal synapses were more intact. We expected that this would facilitate observation of changes in astrocytic mGluR signaling activity following long-term blockade of neuronal APs. The percentage of s.r. astrocytes exhibiting spontaneous Ca^2+^ transients was significantly higher in the TTX-treated slices compared to controls (fig. 3D, p<0.05 in both cases; control: 12.9% ±5.1%; n = 40 cells/8 slices; TTX: 42.1% ±10.1%; n = 30 cells/7 slices). Consistent with our observations in standard bulk-loaded astrocytes, there were no changes observed in the frequency of the spontaneous Ca^2+^ activity in astrocytes (data not shown). Rise time of the spontaneous Ca^2+^ transients for TTX-treated astrocytes was significantly faster compared to the control group (fig. 3E, p<0.01).

Changes in DHPG-evoked responses largely replicated changes observed in the spontaneous activity (fig. 4). While amplitude and response latency of 15 µM DHPG-evoked Ca^2+^ elevations remained consistent between the two groups (fig. 4A and B), the rise time of DHPG-evoked responses was significantly faster in TTX-treated *vs*. control astrocytes (control: n = 32 cells; TTX: n = 27 cells; fig. 4C; p<0.001). To investigate in greater detail possible effects on group I mGluR-evoked response patterns in bolus-loaded astrocytes, DHPG was applied in successive concentrations, 5 and 15 µM. In slices incubated in TTX, astrocytes exhibited more plateau responses to both concentrations of DHPG, while astrocytes from slices incubated in control ACSF exhibited a greater proportion of single-peak or multi-peak responses (fig. 3B; fig. 4D). As summarized in figure 4D, TTX treatment increased the percentage of astrocytes in the population responding to both 5 and 15 µM DHPG, and the response pattern was greatly shifted toward plateau responses at much lower DHPG concentrations (p<0.05 for 15 µM DHPG; control: n = 40 cells/8 slices; TTX: n = 30 cells/7 slices). Both amplitude (fig. 4E) and rise time (fig. 4F) of agonist cocktail-evoked responses remained unchanged, suggesting that the signaling of other Gq GPCRs in s.r. astrocytes was not significantly affected by long-term blockade of neuronal APs. The combination of changes in astrocyte spontaneous and evoked Ca^2+^ activity following incubation in TTX, including a greater percentage of astrocytes in the population exhibiting spontaneous Gq GPCR activity and evoked mGluR responses, a shift toward long-lasting plateau over single peak or multi-peak responses, and a faster rise time for both spontaneous and DHPG evoked Ca^2+^ transients, together suggest that the signaling activity of astrocytic Gq GPCRs, including group I mGluRs, significantly increases after long-term blockade of neuronal APs.

### Long-term Blockade of Neuronal APs Potentiates Gq GPCR-mediated Ca^2+^ Signaling in Astrocyte Microdomains

The above approaches enabled recording of Gq GPCR activity in the astrocyte soma. To record Gq GPCR activity in astrocyte processes, astrocytes were filled with the Ca^2+^ indicator OGB-1 via patch clamp. We first determined what proportion of the astrocyte was filled with Ca^2+^ indicator by comparing the distribution of OGB-1 Ca^2+^ indicator dye to that of Alexa Fluor 568 in the same cells (fig. 5). The boundary of the fine processes of the astrocyte was the same for both channels (fig. 5A). With increased laser power, the signal-to-noise ratio of the cell boundary increased (fig. 5B) but remained similar between channels. New ROIs were placed over “bulk” fine processes that became visible using higher laser power and then the laser power was returned to 0.5% prior to re-application of Gq GPCR agonists. Interestingly, although the baseline fluorescence intensities of neither dye could be distinguished from background, Ca^2+^ elevations were still detected in these ROIs following agonist application (fig. 5C). These data suggest that OGB-1 introduced by patch clamp can effectively monitor Ca^2+^ activity in “bulk” astrocyte fine processes as indicated by overlay with Alexa 568 labeling.

Microdomains of astrocytic Gq GPCR signaling were identified as local spontaneous Ca^2+^ elevations confined to a portion of astrocyte processes [17]. Spontaneous Ca^2+^ transients that propagated over an area ≥75% of the cell including the cell soma were considered “whole-cell” events. Consistent with previous reports, the vast majority of spontaneous Ca^2+^ elevations occurred in microdomains rather than in the soma [31] (fig. 6A and B). The percentage of spontaneous Ca^2+^ transients in both the soma and microdomains was higher in the TTX-treated astrocytes compared to control (fig. 6D, control: n = 10 cells; TTX: n = 11 cells). There was a trend towards a greater number of spontaneous microdomains per astrocyte when normalized to surface area (fig. 6E; p = 0.14, control: n = 29 microdomains/10 cells; TTX: n = 58 microdomains/11 cells). The frequency of spontaneous Ca^2+^ transients remained unchanged after TTX treatment (fig. 6F). Area of propagation and rise time of spontaneous microdomain Ca^2+^ signals were significantly larger and faster, respectively, after incubation in TTX compared to control (fig. 6G and H). Thus, changes in spontaneous astrocyte Ca^2+^ activity are also apparent in astrocyte fine processes after long-term block of neuronal APs.

Furthermore, evoked group I mGluR responses were also enhanced in astrocyte microdomains after long-term incubation of slices in TTX. First, consistent with previous observations, the amplitude of evoked microdomain Ca^2+^ responses remained unchanged (fig. 7A). However, once again the DHPG-evoked microdomain Ca^2+^ rise time was significantly faster in TTX-incubated astrocytes compared to cells in control slices (fig. 7B). Interestingly, after TTX treatment, response latency to 50 µM DHPG was also shorter in microdomains compared to control incubated slices (fig. 7C), possibly because most evoked astrocyte Ca^2+^ elevations initiate in microdomains [23] and then propagate to larger compartments. Finally, there was also a significant shift towards plateau DHPG-evoked Ca^2+^ response patterns in microdomains of TTX-treated astrocytes (fig. 7D). Consistent across all experimental conditions, no significant differences were observed in rise time (fig. 7E) or amplitude (data not shown) of the agonist cocktail-evoked Ca^2+^ responses between TTX-treated and control cells. Overall, data from patch-clamped astrocytes suggest that long-term block of neuronal APs increases the activity of group I mGluRs in astrocyte microdomains.

### The Scaling Effect is Bidirectional: Increased Neuronal Firing Rates Depress Spontaneous and Evoked Group I mGluR Astrocyte Ca^2+^ Transients

If the observed changes in astrocytic Gq GPCR activity occur as a direct consequence of a reduction in neuronal APs, then the effect should be reversible when neuronal AP frequency is significantly elevated. To test this, we compared spontaneous and evoked astrocyte Ca^2+^ elevations in acute hippocampal slices incubated in 5.0 mM [K^+^]_o_ *vs*. 2.5 mM [K^+^]_o_ ACSF (control). We first tested astrocytes bolus-loaded with Ca^2+^ indicator for their spontaneous somatic Gq GPCR activity and responsivity to DHPG (fig. 8A and B). In 5.0 mM [K^+^]_o_ incubated slices, there was a significantly lower percentage of spontaneously active astrocytes (24.9% ±7.8%) compared to control (59.1% ±12.4%) (fig. 8C, p<0.05). In addition, the rise time of spontaneous Ca^2+^ elevations was slower in astrocytes incubated in 5.0 mM [K^+^]_o_ compared to controls (fig. 8D). There was no difference in frequency or amplitude of the spontaneous Ca^2+^ activity (data not shown), consistent with findings after long-term blockade of neuronal APs in TTX.

Three successive concentrations of DHPG (15, 30, and 50 µM) were applied to compare evoked group I mGluR responses between control and 5.0 mM [K^+^]_o_ incubated slices. At 30 and 50 µM DHPG, the rise time of evoked Ca^2+^ elevations in astrocytes incubated in 5.0 mM [K^+^]_o_ was significantly slower compared to control slices (fig. 8E, 30 µM DHPG: p<0.05; 50 µM DHPG: p<0.01, 2.5 mM [K^+^]_o_: n = 29 cells; 5.0 mM [K^+^]_o_: n = 26 cells). There was no difference in rise time of Ca^2+^ responses to agonist cocktail (fig. 8E, p = 0.45, 2.5 mM [K^+^]_o_: n = 52 cells; 5.0 mM [K^+^]_o_: n = 51 cells), suggesting that the effect is primarily due to changes in group I mGluRs. As the concentration of DHPG increased, the percentage of responding astrocytes increased, as expected (fig. 8F, 2.5 mM [K^+^]_o_: n = 52 cells/10 slices; 5.0 mM [K^+^]_o_: n = 51 cells/10 slices). At all DHPG concentrations tested, 5.0 mM [K^+^]_o_ treated astrocytes were less likely to respond, and the pattern of responses shifted toward the weaker, single peak phenotype compared to astrocytes incubated in 2.5 mM [K^+^]_o_ ACSF. There was no difference in the amplitude or latency of DHPG- or agonist cocktail-evoked somatic Ca^2+^ responses in 5.0 mM [K^+^]_o_ treated *vs*. control astrocytes (data not shown).

### Increased Neuronal Firing Rates Also Depress Spontaneous and Group I mGluR-Evoked Ca^2+^ Transients in Astrocyte Microdomains

To determine if the changes in group I mGluR signaling after 5.0 mM [K^+^]_o_ treatment occurs in microdomains, OGB-1 Ca^2+^ indicator dye was delivered into astrocytes via patch clamp. All astrocytes in both conditions exhibited spontaneous Ca^2+^ transients at least once in microdomain(s). In control ACSF, 16.7% of astrocytes had spontaneous somatic Ca^2+^ transients, as opposed to only 9.1% of astrocytes in 5.0 mM [K^+^]_o_ ACSF (fig. 9A). There were no differences in the number of spontaneous microdomains per cell (p = 0.32, fig. 9B). As predicted, the rise time of microdomain spontaneous Ca^2+^ transients in 5.0 mM [K^+^]_o_ treated astrocytes was significantly slower than in control cells (p<0.01, fig. 9C). Consistent with our previous observations, DHPG-evoked Ca^2+^ responses exhibited no difference in amplitude following incubation in 5 mM [K^+^]_o_ (fig. 9D, 2.5 mM [K^+^]_o_: n = 69 microdomains/12 cells; 5.0 mM [K^+^]_o_: n = 37 microdomains/11 cells). However, as expected, the DHPG-evoked rise time was significantly slower following incubation in 5 mM [K^+^]_o_ ACSF compared to the control group (fig. 9E, p<0.05). Also, the latency to evoke group I mGluR-mediated Ca^2+^ responses in microdomains increased significantly after incubation in 5 mM [K^+^]_o_ compared to control (fig. 9F, p<0.001).

To be consistent with experiments in TTX, at the end of each recording, agonist cocktail was applied to verify microdomain responsiveness. As observed throughout our study, there was no change in either rise time or amplitude of agonist cocktail-evoked responses after incubation in 5 mM [K^+^]_o_ (data not shown), suggesting that group I mGluRs are the main astrocytic receptors affected by changes in basal neuronal firing rates (2.5 mM [K^+^]_o_: n = 69 microdomains/12 cells; 5.0 mM [K^+^]_o_: n = 37 microdomains/11 cells). Almost all microdomains that exhibited spontaneous Ca^2+^ transients also responded to agonist cocktail in control conditions. However, only a certain percentage of microdomains responded to 50 µM DHPG after 5.0 mM [K^+^]_o_ incubation (fig. 9G). DHPG at 50 µM normally evokes a Ca^2+^ response that incorporates the entire visible area of the astrocyte, but after long-term increase in neuronal APs, 31.4% of the astrocyte (on avg.) failed to respond to 50 µM DHPG (p<0.001; fig. 9G). In comparison, only 2.8% of each control astrocyte failed to respond to DHPG (2.5 mM [K^+^]_o_: n = 69 microdomains/12 cells; 5.0 mM [K^+^]_o_: n = 51 microdomains/11 cells). This is a very surprising finding that also suggests reduced group I mGluR evoked Ca^2+^ signaling after long-term increase in neuronal APs. Among microdomains that responded after incubation in 5 mM [K^+^]_o_ ACSF, there was a significant shift toward the weaker single-peak phenotype compared to control cells (fig. 9G). Overall, the data suggest that long-term increase in neuronal firing rates reduces astrocytic group I mGluR signaling activity, and that scaling of astrocytic group I mGluR activity is bidirectional.

### Changes in Surface Receptor Activity Account for the Changes in Astrocyte Ca^2+^ Signaling

Because metabotropic receptors are coupled to an intracellular signaling cascade, changes in functional Ca^2+^ signaling may reflect changes in the surface receptors or any number of effector proteins and second messengers. To differentiate among these two possibilities, we chose to use a strain of transgenic mice that express a foreign Gq-coupled metabotropic receptor, the MrgA1R, only in astrocytes [23]. Our earlier work suggested that this receptor incorporates itself into the same membrane compartments and engages the same intracellular signaling machinery as endogenous group I mGluRs [23]. However, because this is a foreign receptor that is not activated by endogenous neurotransmitter released in brain, it should be resistant to long-term changes in neuronal firing rates. Therefore, CA3-CA1 synaptic transmission was manipulated in hippocampal slices from MrgA1^+^ transgenic mice, and Ca^2+^ responses evoked by agonists to endogenous group I mGluRs were compared to those evoked by MrgA1R stimulation in the same slices (fig. 10). As observed in wild type slices, s.r. astrocytes in MrgA1R^+^ slices had spontaneous Ca^2+^ activity mainly in microdomains. MrgA1R^+^ astrocytes responded to 1 µM FMRFa, 4 µM FMRFa, 50 µM tACPD, and agonist cocktail with whole-cell Ca^2+^ elevations (fig. 10A and B). The rise time of the evoked responses was measured as a barometer of changes in Gq GPCR signaling activity based on our earlier data showing that rise time of the Ca^2+^ transients was the most sensitive to changes in receptor activity. Consistent with previous observations, TTX-treated astrocytes had significantly faster rise times of their Ca^2+^ responses to 50 µM tACPD, a group I/group II mGluR agonist, compared to control cells (p<0.05, fig. 10C). These data suggest that 4–6 hr TTX blockade of neuronal APs leads to the same scaling effect on group I mGluR signaling in MrgA1^+^ astrocytes as previously observed in wild type astrocytes. However, in these same astrocytes, there was no difference in rise time of Ca^2+^ responses evoked by either 1 or 4 µM FMRFa (fig. 10C). Furthermore, there were no differences in the MrgA1R response patterns to 1 or 4 µM FMRFa. Two out of 8 control and 2/10 TTX-treated cells responded to 1 µM FMRFa with single peak responses, and one cell from each group responded to 1 µM FMRFa with multi peak responses. The remaining cells (5/8 control, 7/10 TTX) had plateau responses to 1 µM FMRFa. All control and TTX treated cells responded to 4 µM FMRFa with plateau responses. These data provide compelling evidence that the changes observed in group I mGluR Ca^2+^ signaling are due to long-term changes localized to surface group I mGluR activity and not to changes in intracellular Gq GPCR signaling molecules.

## Discussion

It is well-documented that neuronally-released transmitter can stimulate astrocytic Gq GPCRs *in situ* and *in vivo* [1]–[6]. Here we provide evidence for bidirectional scaling of group I mGluR activity in stratum radiatum astrocytes in response to long-term changes in CA3 neuron firing rates. This scaling resembles homeostatic plasticity in neurons and may occur in order to maintain a normal or expected level of excitatory input from neurons. Previous work has established that visible spontaneous and evoked Ca^2+^ signals in passive (non-NG2^+^/non-GluR) hippocampal astrocytes are driven almost exclusively by Gq GPCR-mediated release from internal stores [17], [24], [32], [33]. Therefore, changes in activity levels of astrocytic Gq GPCRs were assayed by recording spontaneous and evoked astrocyte Ca^2+^ transients. Data were replicated using three different, complementary approaches to load astrocytes with Ca^2+^ indicator. After 4–6 hour blockade of action potentials in acute hippocampal slices, we observed the following changes in astrocyte Ca^2+^ signaling: 1) an increased percentage of astrocytes exhibiting spontaneous Ca^2+^ elevations; 2) a faster rise time of spontaneous Ca^2+^ transients; 3) increased response probability to the group I mGluR agonist DHPG; and, 4) a faster rise time (and shorter response latency in microdomains) of evoked mGluR Ca^2+^ responses. These changes were observed in both astrocytic somata and microdomains. Furthermore, 4–6 hr of increased CA3 neuron firing rates in 5 mM [K^+^]_o_ ACSF led to a decrease in the percentage of spontaneously active astrocytes and a significantly slower rise time of both spontaneous Ca^2+^ transients and DHPG-evoked Ca^2+^ responses. In agreement with previous reports of all-or-none astrocytic Gq GPCR responses upon achieving a threshold agonist concentration [30], and lack of correlation of Gq GPCR density to changes in response amplitude [10], no changes were observed in the amplitude of either spontaneous astrocytic Gq GPCR activity or evoked astrocytic mGluR Ca^2+^ elevations.

The observed changes in Ca^2+^ signaling match well with previous studies which have directly assessed the effects of manipulating Gq GPCR expression levels on evoked Ca^2+^ activity. For example, Wang and Thompson [11] found that reduced expression of muscarinic M1 and histamine H1 Gq GPCRs resulted in a reduction in the total number of cells in the population responding to agonist, as well as a longer response latency to agonist. We observed similar changes in astrocytic DHPG responses after 4–6 hr incubation of hippocampal slices in 5 mM [K^+^]_o_. Shao and McCarthy [10], [30] directly compared α1 adrenergic expression levels on phenylephrine-evoked astrocyte Ca^2+^ responses, and found that higher α1 Gq GPCR expression levels correlated directly to an increased percentage of astrocytes in the population responding to agonist. Furthermore, phenylephrine-evoked Ca^2+^ responses had a shorter response latency as expression levels increased. Similar effects were observed in the present study for astrocytic DHPG-evoked responses after 4–6 hr incubation of hippocampal slices in TTX.

Because metabotropic receptors are coupled to intracellular signaling cascades, alterations in Ca^2+^ signaling may be driven by changes in intracellular signaling molecules or the surface receptors. Belair and colleagues [34] recently reported plasticity of metabotropic receptor Ca^2+^ responses in perisynaptic Schwann cells (PSCs) of the frog neuromuscular junction after chronic blockade of postsynaptic nAchRs. Although these researchers did not attempt to differentiate among multiple potential mechanisms of modifications in evoked PSC Ca^2+^ responses, they did observe changes in muscarinic PSC Ca^2+^ response amplitudes, suggestive of a greater amount of Ca^2+^ released from internal stores in these cells [34]. In contrast, we consistently did not find any changes in amplitudes of spontaneous or evoked astrocyte Ca^2+^ events, an indication that coupling to intracellular mechanisms of Ca^2+^ release is not altered by long-term manipulation of neuronal firing rates in our study. To explore this issue more deeply, we performed experiments using hippocampal slices from mice in which astrocytes express a foreign Gq GPCR (the MrgA1R) [23]. As this foreign receptor is not stimulated by neurotransmitter released in brain, it is an ideal tool to distinguish between availability of functional receptors *vs*. coupling to intracellular mechanisms of Ca^2+^ release. In MrgA1R^+^ slices incubated in TTX, agonist-evoked MrgA1R Ca^2+^ elevations were no different from those in MrgA1R^+^ slices incubated in control ACSF. However, evoked group I mGluR responses in the same MrgA1R-expressing astrocytes were significantly faster. As previous evidence suggests that MrgA1 receptors and endogenous group I mGluRs engage the same Gq GPCR signaling molecules [23], these data suggest that the alterations observed in astrocyte Ca^2+^ signaling mainly reflect changes in the surface group I mGluRs.

A characteristic of astrocytic Gq GPCR evoked Ca^2+^ responses is that the pattern is graded from single peak, to oscillatory (“multipeak”), to plateau as the agonist concentration is increased [7], [14], [35], [36]. After incubation in TTX, we found that in comparison to control-incubated slices, DHPG evoked astrocyte Ca^2+^ responses shifted significantly from single peak toward plateau response patterns. This effect was also bi-directional: After increasing CA3 neuronal firing rates in 5 mM [K^+^]_o_ ACSF, the pattern of astrocytic Ca^2+^ responses to DHPG shifted toward single peak responses. The robust effect on response pattern further suggests that the signaling activity of astrocytic group I mGluRs scales up or down following long-term changes in neuronal firing rates. GPCR/Ca^2+^ response patterns may represent distinct “codes” dictating a unique cellular response, conferring a single GPCR subtype with the ability to control various cellular outcomes [7], [14], [36]–[38]. Thus, plasticity of astrocytic Gq GPCRs may result in new signaling codes, leading to long-term changes in astrocyte function.

Spontaneous astrocytic Ca^2+^ activity remains mysterious both in terms of its underlying mechanisms as well as its physiological purpose. While it is clear that these events require IP_3_R-mediated Ca^2+^ release from internal stores [17], [24], it remains to be determined if they are driven by locally released neurotransmitter or by constitutive GPCR activity. A recent report suggests that both action potential-driven and miniature quantal neurotransmitter release contribute to spontaneous astrocyte microdomain Ca^2+^ activity [39]. However, it is also well-documented that GPCRs can signal in the absence of agonist [7], [40]–[43], and astrocytes *in situ* appear to exhibit constitutive Gq GPCR activity. Nett et al. [17] reported that spontaneous somatic astrocyte Ca^2+^ oscillations occur independent of neuronal activity. Furthermore, previous studies *in vitro* established that the amount of constitutive activity is proportional to GPCR expression levels [9], [40], [43]. Therefore, the increased spontaneous Gq GPCR activity in astrocytes after TTX treatment may reflect either increased quantal release of neurotransmitter or increased constitutive GPCR signaling following upregulation of receptor expression levels. This issue is complicated further by the ability of most antagonists (∼ 85% of those tested) to also possess inverse agonist activity [42], [43], preventing dissociation between constitutive *vs*. ligand-mediated receptor activation. Because astrocytes express a wide variety of Gq GPCR subtypes [44], and previous studies have not established which astrocytic Gq GPCRs underlie spontaneous astrocytic Ca^2+^ activity [13], [17], we cannot know for certain which astrocytic receptors may be responsible for changes in spontaneous astrocytic Ca^2+^ transients following manipulation of neuronal firing rates.

Plasticity of astrocytic mGluRs has been demonstrated previously in neurological disorders. For example, mGluRs have been shown to change in reactive astrocytes in models of epilepsy [45]–[47], Taylor-type focal cortical dysplasia [48], and in multiple sclerosis lesions [49]. The present study extends these findings by demonstrating astrocytic Gq GPCR plasticity over a rapid timescale in response to changes in neuronal firing frequency. The plasticity of astrocytic receptors reported here is similar to homeostatic plasticity in neurons and occurs very rapidly, within 4–6 hr in acute hippocampal slices. In neuronal homeostatic plasticity recorded using TTX, “miniature” vesicular release, which remains intact, significantly increases the time course of postsynaptic iGluR scaling by suppression of local dendritic protein synthesis [50], [51]. It is possible that astrocytes are more sensitive to block of AP-mediated release than neurons because astrocytic receptors located on perisynaptic astrocyte processes may have a lower probability to be stimulated by miniature quantal release compared to neuronal postsynaptic receptors. This may especially apply to synapses lacking an associated astrocyte process, a common occurrence in the hippocampus [52], [53].

TTX has been commonly used to induce neuronal homeostatic scaling *in vitro* and *in situ*. Although this is a non-physiological approach, there is evidence to support homeostatic ‘scaling up’ of neuronal receptors following sensory deprivation. For example, Desai et al. [54] reported as little as two days of monocular deprivation scaled up the amplitude of miniature excitatory postsynaptic currents (mEPSCs) in rodent visual cortex in a layer- and age-dependent manner. In the rat olfactory bulb, Tyler et al. [55] found an increased amplitude of AMPA and NMDA mEPSCs following 2 weeks of odor deprivation. Their findings provided a mechanism behind previous work demonstrating increased olfactory bulb sensitivity to odor stimulation after deprivation [56]. Similar “compensatory” scaling effects have also been observed in the cochlear nucleus after deprivation of auditory inputs [57]. Therefore, “depriving” neurons or astrocytes of neuronal inputs using TTX *in vitro* and *in situ* appears to be an appropriate model of compensatory changes based on the effects of sensory deprivation *in vivo*.

The physiological significance of the observed homeostatic-like plasticity of astrocytic mGluRs remains to be determined. An overarching function of astrocytes is homeostasis of the synaptic microenvironment to provide optimal conditions for synaptic signaling. Neuron-to-astrocyte receptor communication may be important for regulating cerebral blood flow [58]–[60] and modulating uptake of K^+^ and glutamate to control neuronal excitability [44], [61], [62]. In addition to these supportive functions, many research groups have reported astrocyte-to-neuron communication via release of neuroactive molecules under certain conditions [63]–[65]. Transcriptome analysis of astrocytes suggests that they are highly secretory cells [66]. Changes in astrocytic Gq GPCR and Ca^2+^ signaling patterns may code for changes in gene transcription [14], [36], [37] to affect the production and secretion of neuromodulators. One candidate is glial TNFα, which has been shown to play diverse roles in brain function including synaptogenesis [67], enhancing release of glutamate from astrocytes [68], [69], as well as contributing to the process of homeostatic plasticity in neurons [70]. Perhaps new response patterns following scaling of astrocytic mGluR activity code for changes in secretion of glial TNFα as part of a compensatory mechanism in attempt to recover normal and expected levels of excitatory synaptic activity in neurons.
